# Nutritional Status, Dietary Practices, and the Double Burden of Malnutrition Among School‐Aged Children in Cameroon: A Systematic Review

**DOI:** 10.1155/jnme/6792104

**Published:** 2026-08-13

**Authors:** Aristide Guillaume Silapeux Kamda, Joseph Hubert Galani Yamdeu, Mercy Achu, Laure Fotso Maptoum, Sylvain Sado, Wilfred Mbacham, Orish Ebere Orisakwe, Roger Ponka, Elie Fokou, Chiara Frazzoli

**Affiliations:** ^1^ Department of Social Economy and Family Management, University of Buea, Buea, Cameroon, ubuea.cm; ^2^ Section of Natural Sciences, School of Sciences, Psychology, Arts and Humanities, Computing, Engineering & Sports, Canterbury Christ Church University, Canterbury CT1 1QU, UK, canterbury.ac.uk; ^3^ Department of Biochemistry, University of Yaoundé I, Yaoundé, Cameroon, uy1.uninet.cm; ^4^ Department of Biotechnology and Pharmacognosy, University of Ebolowa, Ebolowa, Cameroon; ^5^ Department of Microbiology, University of Yaoundé I, Yaoundé, Cameroon, uy1.uninet.cm; ^6^ Advanced Research Centre, European University of Lefke, Lefke Northern Cyprus, TR-10 Mersin, Turkey, eul.edu.tr; ^7^ Department of Agriculture, Livestock and Derived Products, University of Maroua, Maroua, Cameroon, uni-maroua.citi.cm; ^8^ Department of Cardiovascular and Dysmetabolic Diseases, and Aging, Istituto Superiore di Sanità, Rome, Italy, iss.it

**Keywords:** Cameroon, disease prevention, malnutrition, nutrition transition, obesity, overweight, undernutrition

## Abstract

Undernutrition remains highly prevalent among children and adolescents in Cameroon, while increasing rates of overweight and obesity reflect an ongoing nutrition transition and a growing double burden of disease. This systematic review aimed to provide a comprehensive overview of research conducted on nutritional status, dietary intake, and nutrition‐related practices in nursery, primary, and secondary schools in Cameroon. The review was conducted in accordance with the Preferred Reporting Items for Systematic Reviews and Meta‐Analyses (PRISMA) guidelines. Four electronic databases (PubMed, ScienceDirect, Google Scholar, and ResearchGate) were searched for studies published between January 2000 and February 2024. The methodological quality of the included studies was assessed using the Joanna Briggs Institute (JBI) critical appraisal tool. A total of 32 studies met the inclusion criteria. Most studies were conducted in urban areas, particularly in Yaoundé (21.8%), Douala (15.3%), and Bamenda (15.3%), while no studies were identified in the South, East, and Far North Regions. Among primary school children, the prevalence of malnutrition ranged from 9.25% to 30.2%. The highest prevalence of overweight and obesity (38.4%) was observed among secondary school students. Stunting was the most common form of malnutrition among nursery and primary school children. Dietary patterns were characterized by low consumption of fruits and vegetables, with up to 57.3% of children reporting no fruit intake over a 7‐day period. These findings highlight significant regional disparities and the coexistence of undernutrition and overnutrition among school‐aged children in Cameroon. Strengthening nutrition education, improving food environments, and implementing context‐specific interventions are essential to address the double burden of malnutrition without exacerbating existing health risks.

## 1. Introduction

The quantity and quality of food consumed, as well as food safety, play a fundamental role in determining health, well‐being, and overall quality of life. In children, adequate nutrition is essential for optimal physical growth, cognitive development, and psychosocial functioning. Evidence has shown that healthy dietary habits are associated with improved academic performance [1], whereas poor nutrition during childhood can lead to long‐term adverse consequences, including impaired growth, reduced learning capacity, and increased morbidity [2].

Malnutrition encompasses both undernutrition—such as stunting, wasting, underweight, and micronutrient deficiencies—and overnutrition, including overweight and obesity. Regardless of its form, malnutrition remains a major public health concern, particularly in the Global South [3–5]. In recent decades, many countries have experienced a nutrition transition characterized by a shift from traditional diets rich in fiber and complex carbohydrates to more energy‐dense diets high in fats, sugars, and processed foods [6, 7]. This transition has contributed to the rising prevalence of overweight and obesity, even in settings where undernutrition remains widespread. As a result, many populations now face a double burden of malnutrition.

In Cameroon, undernutrition remains highly prevalent, particularly in rural areas, where approximately 29% of children under five are affected by stunting [8, 9]. At the same time, urban areas are experiencing increasing rates of overweight and obesity among school‐aged children and adolescents [9–11]. This dual burden is associated with a growing risk of noncommunicable diseases, including cardiovascular diseases, type 2 diabetes, and hypertension later in life.

The school environment plays a critical role in shaping children’s dietary behaviors and nutritional status. Schools can promote healthy eating through nutrition education and structured interventions; however, they can also expose children to unhealthy food environments, including easy access to energy‐dense, nutrient‐poor foods and beverages [12–14]. In addition, children and adolescents represent particularly vulnerable populations to environmental and nutritional risks, as critical developmental windows increase their susceptibility to harmful exposures and their long‐term health consequences, especially in contexts characterized by socioeconomic deprivation and limited protective infrastructures [13–15]. Environmental factors such as inadequate water, sanitation, and hygiene (WASH) conditions and exposure to infectious agents may further exacerbate malnutrition. Given that dietary habits established during childhood often persist into adulthood, schools represent a key setting for the promotion of healthy behaviors and the prevention of malnutrition in all its forms.

Therefore, the aim of this systematic review is to provide a comprehensive overview of the existing evidence on nutritional status, dietary intake, and nutrition‐related practices among children attending nursery, primary, and secondary schools in Cameroon. By synthesizing available research, this study seeks to identify knowledge gaps and inform the development of effective, context‐specific interventions and policies.

## 2. Methodology

This systematic review was conducted in accordance with the Preferred Reporting Items for Systematic Reviews and Meta‐Analyses (PRISMA) guidelines.

### 2.1. Literature Search

A comprehensive literature search was performed to identify relevant studies published between January 2000 and February 2024. The search was conducted between March and May 2024 using the following electronic databases: PubMed, ScienceDirect, and Google Scholar. In addition, ResearchGate was consulted to identify potentially relevant articles not indexed in traditional databases.

The search strategy combined keywords and Boolean operators related to nutrition, dietary intake, and school‐aged populations in Cameroon. The main search terms included: (“nutrition” OR “diet” OR “nutrient” OR “micronutrient” OR “vitamin” OR “mineral” OR “food”) AND (“intake” OR “consumption” OR “status” OR “practice” OR “intervention” OR “program”) AND (“malnutrition” OR “undernutrition” OR “obesity” OR “overweight” OR “deficiency” OR “anemia”) AND (“nursery school” OR “preschool” OR “primary school” OR “secondary school” OR “pupils” OR “students”) AND (“Cameroon” OR “Cameroonian”).

The search was repeated periodically to ensure inclusion of all eligible studies available up to February 2024. Reference lists of included articles were also screened to identify additional relevant studies.

### 2.2. Inclusion and Exclusion Criteria

The eligibility criteria focused on studies assessing nutritional status and/or school‐based nutrition interventions, including nutrition education, health promotion, physical activity, counseling, and lifestyle management programs among children attending nursery, primary, and secondary schools in Cameroon. No restrictions were applied regarding study design, duration, follow‐up period, or type of intervention. Studies were included if they

• were peer‐reviewed articles published in English or French;
• were conducted in Cameroon;
• involved children enrolled in nursery, primary, or secondary schools;
• reported quantitative/qualitative data on nutritional status, dietary habits, physical activity, or the impact of school‐based nutrition interventions.

Studies were excluded if they

• consisted of gray literature, preprints, dissertations, conference abstracts, or unpublished manuscripts;
• focused on populations outside the school setting (e.g., household‐, hospital‐, or community‐based interventions);
• targeted higher education students;
• addressed exclusively therapeutic interventions for diagnosed nutritional diseases or eating disorders;
• focused solely on pharmaceutical or plant‐based treatments without relevance to school nutrition.

### 2.3. Study Selection and Data Screening

All identified records were initially screened by one reviewer (A.G.S.K.) based on titles and abstracts. Studies that clearly did not meet the eligibility criteria were excluded at this stage. When abstracts provided insufficient information, full‐text articles were retrieved and assessed.

The quality and relevance of the selected studies were independently evaluated by three reviewers (A.G.S.K., G.Y.J.H., and C.F.). In addition, reference lists of eligible articles were manually screened to identify further relevant studies. Discrepancies between reviewers were resolved through discussion and consensus.

All records identified through the database search were imported and screened in three stages: title screening, abstract screening, and full‐text review.

**FIGURE 1:**
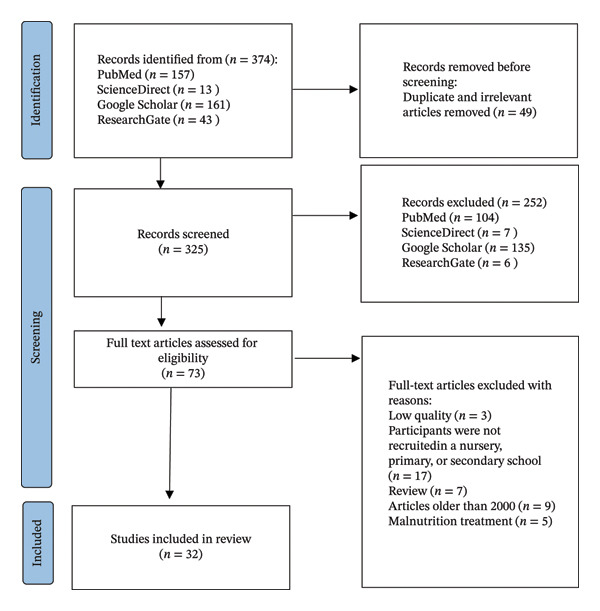
Literature search and identification process of potential references.

The study selection process is summarized in a PRISMA flow diagram (Figure 1).

### 2.4. Data Extraction

Data were extracted independently by the reviewers using a standardized data extraction form. The following information was collected from each included study:

• Author(s) and year of publication
• Study location and setting
• Study design
• Sample size and participants’ age
• Type of school (nursery, primary, or secondary)
• Variables measured (e.g., anthropometric indicators, dietary intake, and socioeconomic factors)
• Data collection methods and instruments
• Key findings relevant to the review objectives

To ensure accuracy and consistency, extracted data were cross‐checked by an additional reviewer (C.F.).

### 2.5. Quality Assessment

The methodological quality of the included studies was assessed using the Joanna Briggs Institute (JBI) critical appraisal tool for analytical cross‐sectional studies [16].

The tool evaluates key domains including selection bias, measurement validity, confounding factors, and statistical analysis. Each study was scored based on eight criteria, with one point assigned for each criterion met [17, 18].

Studies were classified according to their total score as follows:

• **High quality:** 6–8 points
• **Moderate quality:** 4–5 points
• **Low quality:** 0–3 points

Quality assessment was conducted independently by two reviewers (A.G.S.K. and G.Y.J.H.). Any disagreements were resolved through discussion and consensus.

## 3. Results

### 3.1. Study Selection

The initial database search identified a total of 374 records (PubMed: 157; ScienceDirect: 13; Google Scholar: 161; ResearchGate: 43). After removing duplicates and clearly irrelevant records (*n* = 49), 325 articles remained for abstract screening.

Following abstract screening, 252 articles were excluded. The full texts of the remaining 73 studies were assessed for eligibility, leading to the exclusion of 41 articles for the following reasons: low methodological quality (*n* = 3), nonschool‐based populations (*n* = 20), review articles (*n* = 7), publication before 2000 (*n* = 9), and focus on therapeutic interventions (*n* = 5).

A total of 32 studies met the inclusion criteria and were included in the final analysis. The study selection process is presented in Figure 1.

### 3.2. Characteristics of Included Studies

The 32 included studies were categorized into three main areas: nutritional status (*n* = 24), nutritional assessment (*n* = 6), and nutrition influencers and actions (n = 5).

Most studies (84.4%) employed a cross‐sectional design, while three were quasi‐experimental and one used qualitative methods.

**FIGURE 2:**
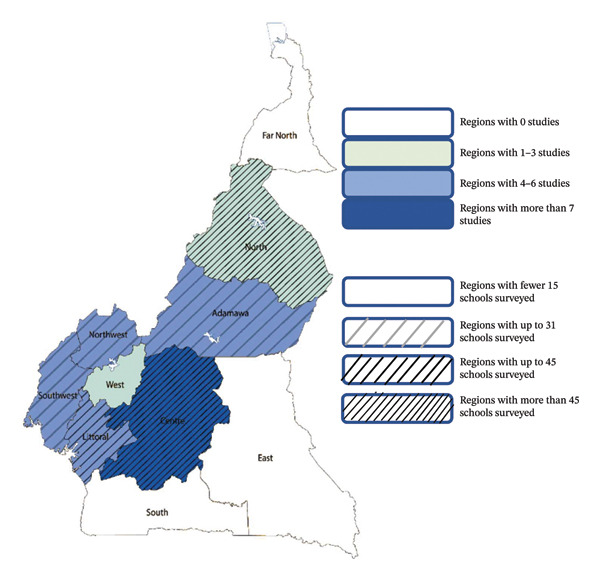
Mapping of nutrition studies conducted in the 10 regions of Cameroon.

Geographically, the majority of studies were conducted in the Centre (25%), Littoral (15.3%), Northwest (15.3%), and Southwest (12.5%) Regions (Figure 2). More than half of the studies were carried out in urban areas, particularly in Yaoundé (21.8%), Douala (15.3%), and Bamenda (15.3%). No studies were identified in the South, East, and Far North Regions.

Overall, the studies covered 260 schools, including 19 nurseries (7.3%), 184 primary schools (70.7%), and 57 secondary schools (22%). Most schools were public (75.7%). The total sample size across all studies was 18,113 students, aged between 2 and 18 years.

Anthropometric measurements (e.g., BMI, waist circumference, mid‐upper arm circumference, and skinfold thickness) and sociodemographic data were the most commonly assessed variables. Data collection methods primarily included standardized measurement tools and structured questionnaires.

Based on the JBI quality assessment, 17 studies were classified as high quality and 14 as moderate quality. Studies rated as low quality were excluded from the analysis.

Table 1 summarizes the characteristics of the reviewed studies.

**TABLE 1:** Features of the studies covered in the review.







*Note:* Studies are ordered by region and by year. Categories (nutritional status, nutritional assessment, nutrition influencers and actions) are highlighted in different colors; different colors are applied to different focuses of the studies, that is, malnutrition risk factors, socioeconomic factors, or relation with infectious agents.

### 3.3. Nutritional Status and Associated Factors

Table 2 summarizes key findings on nutritional status, and risk and protective factors. Nutritional status varied across school levels, regions, and socioeconomic groups, reflecting the coexistence of undernutrition and overnutrition among school‐aged children in Cameroon.

**TABLE 2:** Nutritional status and key factors.

School level	Malnutrition	‐Prevalence	Risk factors	Protective factors	References
		‐Range			
		‐No. of studies			
		‐Prevalence of boys/girls			
		‐High/low SES			
Nursery	Stunting	42 ± 18.34% [16.4%–55%]	High household size, poverty, low education, low resting energy expenditure, farming mother	Low household size, intake of animal protein, eating dairy products	[29, 32, 48]
		3 studies			
		22.2% boys			
		10.7% girls			
	Underweight	14.63 ± 13.58% [5.2%–30.2%]	High household size farming mother	Low household size	[29, 32, 48]
		3 studies			
		16.4% boys			
		7.4% girls			
	Wasting	7.52 ± 4.53% [3.2%–13.8%]	High household size farming mother	Low household size	[29, 32, 48]
		3 studies			
	Overweight	1%	Low household size, high birth weight, resting energy expenditure, low education	High household size	[29, 48]
		1 study			
	Malnutrition	19.2%	High household size	Low household size	[48]

Primary school	Stunting	14.4 ± 8.45% [1.7%–28.6%]	Male, public school, high family size, illiterate mother	Female, private school	[9, 22–24, 37, 38, 41, 43]
		8 studies			
		18.9 ± 13.71% boys; [9.2%–28.6%]			
		2 studies			
		10.85 ± 11.95% girls [2.4%–19.3%]			
		2 studies			
		21.4% (public)			
		1.7% (private)			
	Underweight	4 ± 2.25% [0.7%–6%]	Public school, male, low income	Catholic school, female, higher income	[9, 20, 22, 23, 26, 39, 41, 45]
		9 studies			
		5.5% (boys)			
		4.8% (girls)			
		6.0% (public)			
		0.6% (private)			
		23.8% (high SES); 22.6% (low SES)			
	Wasting	2.18 ± 3.60% [0.3%–9.5%]	Female	Male	[23, 37, 39, 42, 45]
		6 studies			
		1.6% (boys)			
		2.9% (girls)			
		0.00% (public)			
		3.4% (private)			
	Overweight	14.36 ± 9.90 [1.6%–29.9%]	High SES, high birth weight, family history of overweight	Low SES, low birth weight, being born at term	[9, 23, 24, 26, 31, 38, 40, 45]
		6 studies			
		13% boys			
		12.6% girls			
		9.5 (high SES)			
		6.53 (low SES)			
	Malnutrition	24.9 ± 9.01 [19.8%–35.6%]	Male, Catholic, low SES, high birth weight, intestinal helminth infections	High SES, low birth weight	19, 42, 43, 44, 45, 46, 47]
		6 studies			
		21.8% boys			
		20.72% girls			
		22.0% (public)			
		26.2% (private)			

Secondary school	Stunting	15 ± 0.1% boys [14%–15% boys]	Low and moderate SES, boys	High SES, female	[31, 36]
		2 studies			
		5.5% boys			
		5% girls			
		5% (high SES)			
		12% (low SES)			
	Underweight	5.5 ± 0.7% boys [5%–6% boys]	Low and moderate SES, boys	High SES, girls	[31, 36]
		2 studies			
		1.5% girls			
		1% (high SES)			
		3% (low SES)			
	Overweight	14.25 ± 0.95% [13.6%–14.9%]	High SES, girls	Low SES, boys	[19, 27, 31, 36]
		2 studies			
		4%(boys)			
		14% (girls)			
		8% (low SES)			
		9% (high SES)			
	Obesity	29.92 ± 17.04% [4.6%–35.5%]	Girls, physical inactivity, hyperglycemia, high SES, girls	Boys, low SES, boys	[19, 21, 27, 36]
		4 studies			

*Note:* SES: socioeconomic status.

#### 3.3.1. Nursery Schools

The findings highlight a complex nutritional landscape among nursery school children characterized by the simultaneous presence of undernutrition (stunting, underweight, wasting) and overnutrition, commonly referred to as the double burden of malnutrition. While stunting and underweight persist in many settings, overweight individuals were scarce and limited to specific urban settings such as Yaoundé. Recurrent risk factors for undernutrition included large household size, low maternal education, and limited dietary diversity, while protective factors included higher intake of animal‐source foods and dairy products.

#### 3.3.2. Primary Schools

Research highlights a significant double burden of malnutrition where undernutrition (stunting, wasting, and thinness) coexists with rising rates of overweight and obesity in the same population. Boys were more affected by stunting and underweight, while wasting was slightly higher among girls. Overweight prevalence reached 14.4%, indicating the emergence of overnutrition at this level. Boys and children from lower socioeconomic backgrounds were more affected by undernutrition, while overweight was more common among children from higher socioeconomic status, born with higher birth weight, and those attending private schools.

Reported risk factors included low household income, large family size, and parasitic infections, whereas higher socioeconomic status and improved dietary conditions were associated with better nutritional outcomes.

#### 3.3.3. Secondary Schools

Among secondary school students, overweight (14.3%) and obesity (29.9%) were the most prevalent forms of malnutrition. These conditions were more common among girls and students from higher socioeconomic backgrounds.

In contrast, undernutrition (stunting and underweight) persisted among boys and students from lower socioeconomic groups. Physical inactivity, high‐calorie diets, and metabolic risk factors (e.g., hyperglycemia) were identified as key contributors to overweight and obesity.

### 3.4. Food Intake and Nutritional Practices

Dietary patterns and nutritional practices varied significantly across regions and school levels (Table 3).

**TABLE 3:** Nutritional practices and food intakes, nutritional knowledge, and physical activities.

School level	Nutritional practice	Food intake	References
Nursery	The mean dietary diversity score for stunted obese children was significantly lower than the one for obese children	‐ 50.7% of the children consumed mostly cereals and tubers ‐ 7.4% consumed meat, fish, and eggs ‐ 3%, 7% consumed milk and milk products. ‐ 3.2% consumed vegetables and fruits.	[29, 31]
	‐ Girls were less active and spent less time doing moderate‐to‐vigorous activities ‐ Stunted obese and stunted children spent more time in sedentary activities than obese children and less time in moderate‐to‐vigorous activities than nonstunted, non‐obese children	Stunted children are fewer to consume dairy products than nonstunted children	[29]

Primary school	‐ 63.5% of students have at least three meals per day. ‐ Students eat diversified foods but have low intake of minerals ‐ Displaced students (56%) in the West Region have only 2 meals a day ‐ 23% of displaced primary students had very low nutrition literacy ‐ 27% had moderate nutrition literacy.	‐ The average consumption of carbohydrates, lipids, and proteins was 280.1, 24, and 39.9 g/day, respectively. ‐ The daily energy intake of the boys ranged between 89.5% and 100.6% of their energy requirement, and that of girls ranged between 100.9% and 114.1% ‐ The average consumption of calcium was 430.9 mg/day	[23, 24, 35]
	Nutritional education permits improvements in food choices and increase the proportion of students who consumed fresh fruits, enriched cakes, and doughnuts made with local crops	‐ The average daily protein intake was over 50% for all children. ‐ The daily intakes of Ca, Mg, K, Zn, and Fe were lower than the daily requirement values. ‐ The intake of minerals was low.	[23, 25]
	‐ 35.8% of students have poor nutrition knowledge ‐ 27% have average nutrition knowledge ‐ 2.9% have excellent nutrition knowledge ‐ Displaced children ate mainly cereal flour and vegetables ‐ 56% of students ate only 2 times in a day. ‐ Physical activity had a negative correlation with overweight/obesity and the thickness of the triceps skin fold ‐ Fatness and triceps skinfold thickness had a negative correlation with physical activity.	‐ Only 3% of the pupils consumed animal products more than 7 times in a week ‐ Up to 57.3% and 36% of children never ate fruits and vegetables within the period of seven days ‐ Only 32.3% and 29.7% consumed daily dark green leafy vegetables and meat and fish, respectively ‐ 61.2% were capable of satisfying their RDI for energy, whereas only about half of them were able to meet the RDI for protein	[35, 38]

Secondary school	‐ Urban teenagers’ daily activities are watching television, listening to music, and washing. ‐ Rural teenager’s daily activities are farming, carrying water, dishing, washing, and cooking. ‐ Female students spent more time on low‐intensity activities such as doing homework, household activities, watching television, going by car, and praying, while male students spent more time doing sports activities. ‐ Students who exercise 02 days/week had 14% less risk of being obese as compared to those who do not practice sports (CI: 0.31 to 2.15; *p*‐value 0.70). ‐ The usage of vehicles and bikes to go to school compared to walking increases the odds of being overweight by 74% (OR: 0.26; CI: 0.14–0.64; *p* = 0.0026).	‐ Students consuming alcohol have high risk to become obese. ‐ Consumption of pastry increased by 43% the odds of being obese compared to the consumption of “eru” and “fufu” (CI: 0.30 to 6.31; *p*‐value 0.63). ‐ The majority of students admitted to consume alcohol ‐ The rate of alcohol consumption was 65.8%; the rate for males (73.1%) was higher than the rate for females (62.9%) ‐ The rate of tobacco consumption is 3.6%. The value is higher for males (7.1%) compared to females (2.2%).	[19, 20, 27, 30, 34]
	More than 30% of the adolescents skipped breakfast in the urban area.	7% of students with high SES ate milk and dairy products compared to 2% and 0.8% from rural areas or low SES, respectively	[30, 34]
	67% girls reported eating in‐between meals compared to boys (41%)	Over 55% of the students had a protein intake below 10% of the energy (E%).	[30, 34]
	‐ Boys and the adolescents with low SES reported a higher energy expenditure and physical activity level than girls and the adolescents with high SES ‐ About 58.3% engaged in weekly physical activity, and 49.8% spent at least 3 h per day in front of screens.	‐ Eggs and milk products were consumed five times more by the urban than the rural students. ‐ A higher rate of male students compared to female had an intake of vitamin A below the RDA.	[30, 34]
	Children exposed to video treatment improved their nutrition knowledge scores than students who received traditional classroom instruction	Junk food was more often consumed by the students with high SES (24.4%) compared to those with low SES(17.9%) or from rural area (6.7%)	[30, 34]
	The more educated parents are, the better is the nutritional status of their kids	Vitamin B1 intake was significantly higher among boys (0.7%) than girls (0.6%)	[30, 34, 45, 47]
	Girls are mainly involved on low‐intensity activities such as doing homework, household activities, sitting, watching television, and praying, while boys spend more time doing other sports activities	‐ Three point seven, eleven, and eleven point height percent of urban students with low SES ate eggs, fruits and meat and fish compared to 0.6%, 8.9%, and 4.5% from rural area, respectively ‐ Teenagers with low SES were more likely to be below the recommendations for lipids and vitamins B than those with high SES.	[30, 34]

*Note:* SES: socioeconomic status.

Abbreviations: RDA, recommended dietary allowance; RDI, reference daily intake.

#### 3.4.1. Nursery Level

The finding indicates that the double burden of malnutrition in children is driven by a nutrition transition characterized by high intake of processed, sugar‐dense foods such as cereals and tubers alongside insufficient micronutrients with low consumption of animal‐source foods, fruits, vegetables, and dairy. Limited dietary diversity was associated with both stunting and combined stunting‐obesity conditions.

#### 3.4.2. Primary Level

Most primary school children reported consuming two to three meals per day; however, dietary quality was often inadequate.

Low intake of fruits and vegetables was common, with up to 57.3% and 36% of children reporting no fruit and vegetable consumption within a 7‐day period. Animal‐source foods were rarely consumed in some regions, particularly among internally displaced populations.

Although energy intake was generally adequate for a majority of students, intake of essential micronutrients (e.g., calcium, iron, and zinc) was often below recommended levels.

Nutrition education interventions demonstrated positive effects, including improved dietary knowledge, increased consumption of healthy foods, and reduced intake of energy‐dense snacks. Physical activity was inversely associated with overweight and adiposity indicators.

#### 3.4.3. Secondary Level

Nutritional practices are largely characterized by a low intake of dietary fiber, micronutrients, fruits, and vegetables, contrasted with high consumption of energy‐dense, nutrient‐poor, ultra‐processed foods. Among adolescents, dietary behaviors were strongly influenced by socioeconomic status, gender, and urban‐rural context. Urban students were more likely to consume processed and energy‐dense foods, while rural students reported higher levels of physical activity. Sedentary behaviors, including screen time, were common, particularly in urban settings.

Skipping meals, especially breakfast, and frequent snacking were also reported. Alcohol consumption was prevalent among secondary school students, with higher rates observed among males. Regular physical activity was associated with a reduced risk of overweight and obesity, while active commuting (e.g., walking to school) was linked to better weight outcomes.

## 4. Discussion

This systematic review provides a comprehensive overview of the nutritional status, dietary patterns, and nutrition‐related practices among school‐aged children in Cameroon. The findings highlight the coexistence of undernutrition and overnutrition, reflecting a clear double burden of malnutrition across nursery, primary, and secondary school levels.

### 4.1. Overall Burden and Geographic Disparities of Malnutrition

Stunting was the most prevalent form of malnutrition among nursery, primary, and secondary school students in Cameroon, followed by underweight and wasting [23, 42, 43, 48]. This reflects a high burden of chronic undernutrition across school‐aged populations. However, prevalence varies substantially across regions and between rural and urban settings, while increasing overweight and obesity in secondary schools indicate an ongoing nutrition transition.

A major limitation in the literature is the absence of school‐based studies in the East, South, and Far North Regions, despite these areas reporting some of the highest national rates of child malnutrition (32.8% in the East and 36.4% in the Far North) [49, 50]. In contrast, more than half of the studies were conducted in urban centers such as Yaoundé, Douala, and Bamenda, even though malnutrition is generally more prevalent in rural areas [51]. This indicates a geographical research bias likely driven by logistical, administrative, and security constraints. In fact, the Far North Region of Cameroon is drastically affected by drought, and this has been exacerbated by climate change in recent years, with a subsequent negative impact on food security among the population [52]. Added to that, this region is regularly targeted by attacks of the Nigeria‐based Boko Haram terrorist group. The terrorist attacks led to internal migration of populations, making them vulnerable to malnutrition. Besides, due to the security constraints, researchers do not feel safe to run field work in this region, hence the absence of school‐based studies in the Far North Region. The East and South Regions of the country are relatively safer, but these areas have a low population and a low population density. Also, road infrastructures are not well developed in these regions, which are covered by a dense tropical forest, making it difficult for researchers to travel there.

### 4.2. Dietary Patterns, Physical Activity, and Lifestyle Behaviors

Evidence highlights important differences in dietary intake and physical activity according to nutritional status and sociodemographic characteristics. Dietary diversity is also reduced among stunted children, who consume fewer food groups, including dairy products, compared to nonstunted peers [29]. Although some children meet energy requirements, diets remain nutritionally imbalanced, particularly regarding micronutrients [23, 35]. In primary schools, internally displaced children reported lower meal frequency and monotonous diets based mainly on cereals and vegetables. Urban children, although more likely to meet energy needs, still showed micronutrient deficiencies, particularly calcium, magnesium, zinc, and iron [23]. These observations suggest that stunting is driven by the poor quality of children’s diet rather than just a shortage of food volume. While stunted children may eat enough starchy staple foods to meet their basic daily calorie needs, they lack access to a wide variety of nutrient‐dense food groups such as dairy, eggs, fruits, and vegetables. Due to this nutritional imbalance or hidden hunger, the body receives enough fuel to prevent starvation but lacks the essential vitamins and minerals (like iron, zinc, and vitamin A) required for healthy bone growth, brain development, and immune function [53]. Therefore, preventing stunting in this population requires a focus on nutrient richness and dietary diversity, not just stomach‐filling and calorie‐rich foods. Among adolescents, alcohol and tobacco use were more frequent in males than females, potentially contributing to overweight and obesity, especially in urban contexts where dietary and lifestyle transitions are more pronounced [20].

### 4.3. Socioeconomic and Sociodemographic Determinants

Malnutrition is strongly associated with age, socioeconomic status, living environment, and sex. Boys and children aged 10–14 years are more frequently affected by stunting and thinness [30, 37, 43, 44], likely due to higher energy requirements and greater physical activity [19, 22, 54, 55]. In contrast, overweight and obesity are more common in girls, possibly due to lower physical activity and greater exposure to household food environments [55]. The pooled prevalence of malnutrition among primary school children (24.9%) is within the regional range, higher than Ghana (21.5%) [56–59] and Ethiopia (21.2%) [60], but lower than Burkina Faso (35.1%) [61] and Sudan [55].

The prevalence of overweight and obesity in Cameroon, currently estimated at 14.4%, is notably higher than the corresponding figures for Ethiopia at 11.9% and Nigeria at 4.9% [60, 62]. This variation in obesity rates among these countries may not stem solely from differences in dietary habits. Still, it may also be influenced by the classification systems used to measure body mass index (BMI). Most studies on this topic have utilized the World Health Organization (WHO) criteria, while others have adopted the Centers for Disease Control and Prevention (CDC) standards [56]. The discrepancies in BMI categorization could contribute to the observed differences in obesity rates across these countries.

In urban regions of Cameroon, the dietary landscape is increasingly characterized by the consumption of energy‐dense foods, which are often high in sugars and fats, alongside a rise in sedentary lifestyle habits. These factors are exacerbated by the proliferation of processed food products, readily available in urban markets, which contribute to an elevated risk of overweight and obesity [20, 22, 33]. The urban population tends to experience rapid changes in lifestyle associated with urbanization, which often includes reduced physical activity and the adoption of less healthy eating patterns.

Conversely, rural areas in Cameroon face significant challenges related to food insecurity, which manifests as insufficient access to nutritious food, leading to low dietary diversity and undernutrition among many communities [33, 38]. This juxtaposition highlights a critical aspect of the nutritional transition occurring within the country, where different areas experience contrasting nutritional challenges. While urban populations grapple with the health implications of excess caloric intake and obesity, rural populations may struggle with the consequences of malnutrition and undernutrition.

These patterns highlighted in the current study are vital to addressing the dual burden of malnutrition that exists among school‐aged children in Cameroon. Policymakers and public health officials must develop targeted interventions that address both overweight and obesity in urban settings while simultaneously ensuring that rural populations gain access to a diverse and nutritious diet. This multifaceted approach is essential for improving overall health outcomes and achieving a balance in nutritional health across the nation’s diverse geographic and socioeconomic landscapes.

### 4.4. Infectious Diseases and Environmental Determinants

Malnutrition is strongly correlated with infectious diseases, including intestinal helminths, malaria, and food‐borne infections [24, 41, 44, 46]. These diseases significantly impair nutrient absorption by the body and elevate the metabolic demands placed on individuals, particularly vulnerable populations such as children. This interaction creates a vicious cycle, where infections exacerbate malnutrition, and malnutrition, in turn, heightens susceptibility to further infections [14, 61, 63, 64]. In children, polyparasitism (where multiple parasitic infections coexist) has been linked to increased rates of acute malnutrition [46]. Specifically, helminth infections can lead to nutritional deficiencies by interfering with the body’s ability to efficiently utilize consumed nutrients [44]. Additionally, factors such as unsafe drinking water, poor sanitation, and inadequate hygiene practices exacerbate the burden of these health issues, often leading to a higher prevalence of both infectious diseases and malnutrition [20, 24, 36, 43]. Therefore, improving water quality and sanitation infrastructure is crucial. It is recommended to implement effective interventions among school‐aged children in the affected areas to help break the cycle of infection and undernutrition [65, 66].

### 4.5. Structural Determinants: Poverty, Food Systems, and Nutrition Knowledge

Malnutrition is intricately linked to the intertwined issues of poverty and limited access to essential services, such as education, healthcare, and adequate dietary options [67, 68]. Households with low income often resort to purchasing inexpensive yet nutrient‐poor foods, which can lead to increased vulnerability to various forms of malnutrition, including both undernutrition and diet‐related noncommunicable diseases [68]. This risk is particularly pronounced in rural areas, where access to nutritious food and healthcare services is often more limited [33, 38]. Dietary behaviors within these households are significantly influenced by factors such as parental education, household practices, and the availability of school‐based nutrition education [22, 24, 29, 30, 35, 45]. Research has shown that low levels of nutritional knowledge are often associated with unhealthy eating patterns, irregular meal times, and a lack of variety in diets [19, 25, 27, 35]. In contrast, nutrition education initiatives have demonstrated effectiveness in improving dietary choices and reducing the incidence of both undernutrition and obesity [69, 70]. Consequently, there is potential to foster healthier eating habits by enhancing nutritional knowledge among parents and communities of school‐aged children in Cameroon. This could ultimately mitigate the adverse effects of malnutrition and promote better overall health outcomes.

### 4.6. Integrated Interpretation and Public Health Implications

The coexistence of undernutrition, overweight, and obesity, combined with infectious diseases and environmental constraints, highlights the complexity of the nutritional situation among school‐aged children in Cameroon. These findings reflect ongoing epidemiological and nutrition transitions driven by urbanization, lifestyle changes, and socioeconomic inequalities [5, 58, 71].

Integrated public health strategies addressing undernutrition, obesity, infectious diseases, and environmental factors are urgently needed. School‐based interventions combining nutrition education, hygiene promotion, and physical activity are particularly relevant.

Future research should prioritize underrepresented regions and adopt longitudinal and intervention designs. Strengthening school‐based surveillance and intersectoral collaboration between health and education systems is essential for evidence‐based policy and sustainable improvement.

### 4.7. Strengths and Limitations of This Study

This review provides a comprehensive synthesis of available evidence on school‐based nutrition in Cameroon, covering nutritional status, dietary patterns, and intervention approaches. The use of standardized methodological frameworks, including PRISMA guidelines and JBI quality appraisal, strengthens the reliability and transparency of the findings.

However, several limitations should be considered. Most included studies were cross‐sectional, which limits causal inference. In addition, geographical coverage was uneven, with key regions underrepresented, potentially introducing selection bias. Methodological heterogeneity in study designs, measurement tools, and outcome definitions may also affect comparability across studies. Finally, the exclusion of gray literature may have resulted in the omission of relevant evidence.

## 5. Conclusion

Malnutrition remains a significant public health concern among school‐aged children in Cameroon, affecting all educational levels from nursery to secondary school. The findings of this review highlight the coexistence of undernutrition and overnutrition, reflecting a persistent and complex double burden of malnutrition across both urban and rural settings.

Nutritional status was found to be strongly associated with socioeconomic conditions, demographic characteristics, dietary behaviors, and exposure to infectious diseases. Despite their limited number, school‐based nutrition interventions demonstrated positive effects on dietary practices and health outcomes, underscoring the potential of schools as key platforms for preventive action.

Addressing malnutrition in this context requires integrated and multisectoral approaches that combine nutrition education, promotion of healthy dietary behaviors, increased physical activity, and improved access to safe WASH. Importantly, policies and interventions should be carefully designed to ensure that efforts to reduce undernutrition do not inadvertently contribute to rising rates of overweight, obesity, and diet‐related noncommunicable diseases.

Future research should focus on underrepresented regions, particularly those with high levels of malnutrition, and prioritize longitudinal and intervention‐based studies. Strengthening collaboration between the health and education sectors will be essential to support evidence‐based policies and sustainable improvements in child nutrition in Cameroon.

## Author Contributions

A.G.S.K. led the study, including data collection, screening, data extraction, analysis, and drafting of the manuscript. C.F. conceived the study, contributed to the study design and methodology, supervised the research, and critically revised the manuscript. J.H.G.Y. contributed to the screening of titles and abstracts. J.H.G.Y., M.A., and L.F.M. contributed to full‐text screening and data extraction. E.F., W.M., S.S., O.E.O., and R.P. contributed to data interpretation and critically revised the manuscript.

## Funding

No funding was received for this manuscript.

## Disclosure

All authors read and approved the final manuscript.

## Conflicts of Interest

The authors declare no conflicts of interest.

## Data Availability

The datasets generated during and/or analyzed during the current study are available from the corresponding author on reasonable request.

## References

[1] Mukhamedzhanov E. , Tsitsurin V. , Zhakiyanova Z. , Akhmetova B. , and Tarjibayeva S. , The Effect of Nutrition Education on Nutritional Behavior, Academic and Sports Achievement and Attitudes, IJEMST. (2023) 11, no. 2, 358–374, 10.46328/ijemst.3133.

[2] Beluska-Turkan K. , Korczak R. , Hartel B. et al., Nutritional Gaps and Supplementation in the First 1000 Days, Nutrients. (2019) 11, no. 12, 10.3390/nu11122891. PMC6949907 31783636

[3] Webb P. , Stordalen G. A. , Singh S. , Wijesinha-Bettoni R. , Shetty P. , and Lartey A. , Hunger and Malnutrition in the 21st Century, BMJ. (2018) 361, 10.1136/bmj.k2238. PMC5996965 29898884

[4] United Nations Children’s Fund , Undernourished and Overlooked: A Global Nutrition Crisis in Adolescent Girls and Women, UNICEF Child Nutrition Report Series, 2022, 2023, UNICEF, New York, NY.

[5] Frazzoli C. and Mantovani A. , Toxicological Risk Factors in the Burden of Malnutrition: The Case of Nutrition (And Risk) Transition in Sub-Saharan Africa, Food and Chemical Toxicology. (2020) 146, 10.1016/j.fct.2020.111789. 33011353

[6] Lobstein T. , Baur L. , and Uauy R. , Obesity in Children and Young People: A Crisis in Public Health, Obesity Reviews. (2004) 5, no. 1, 4–104, 10.1111/j.1467-789X.00133.x. 15096099

[7] Popkin B. M. and Gordon-Larsen P. , The Nutrition Transition: Worldwide Obesity Dynamics and Their Determinants, International Journal of Obesity and Related Metabolic Disorders. (2004) 28, no. 3, S2–S9, 10.1038/sj.ijo.0802804. 15543214

[8] National Institute of Statistics (NIS) and ICF , 2018 Cameroon Demographic and Health Survey, Yaounde, Cameroon and Rockville, 2020, NIS and ICF, Maryland, USA.

[9] Cedric F. W. P. , Oben J. E. , and Cianflone K. , Prevalence of Overweight, Obesity, and Thinness in Cameroon Urban Children and Adolescents, Journal of Obesity. (2013) 2013, 737592, 10.1155/2013/737592. 23862056 PMC3703727

[10] Choukem S. P. , Kamdeu-Chedeu J. , Leary S. D. et al., Overweight and Obesity in Children Aged 3-13 Years in Urban Cameroon: A Cross-Sectional Study of Prevalence and Association With Socio-Economic Status, BMC Obesity. (2017) 4, no. 1, 10.1186/s40608-017-0146-4. PMC5286775 28163924

[11] Juonala M. , Magnussen C. G. , Berenson G. S. et al., Childhood Adiposity, Adult Adiposity, and Cardiovascular Risk Factors, New England Journal of Medicine. (2011) 365, no. 20, 1876–1885, 10.1056/NEJMoa1010112. 22087679

[12] Pineda E. , Bascunan J. , and Sassi F. , Improving the School Food Environment for the Prevention of Childhood Obesity: What Works and what Doesn’t, Obesity Reviews. (2021) 22, no. 2, 10.1111/obr.13176. 33462933

[13] Cohen Hubal E. A. , de Wet T. , Du Toit L. et al., Identifying Important Life Stages for Monitoring and Assessing Risks From Exposures to Environmental Contaminants: Results of a World Health Organization Review, Regulatory Toxicology and Pharmacology. (2014) 69, no. 1, 113–124, 10.1016/j.yrtph.2013.09.008. 24099754 PMC5355211

[14] Nwanaforo E. , Obasi C. N. , Frazzoli C. , Bede-Ojimadu O. , and Orisakwe O. E. , Exposure to Environmental Pollutants and Risk of Diarrhea: A Systematic Review, Environmental Health Insights. (2024) 18, 10.1177/11786302241304539. PMC11608449 39619770

[15] Frazzoli C. , The Vulnerable and the Susceptible: The Weight of *Evidenza* to Stop Exploiting Activities Generating Toxic Exposures in Unprotected and Deprived Countries, Journal of Global Health. (2021) 11, 10.7189/jogh.11.03046. PMC8005273 33828831

[16] Moola S. , Munn Z. , Tufanaru C. et al., Aromataris E. and Munn Z. , Chapter 7: Systematic Reviews of Etiology and Risk, JBI Manual for Evidence Synthesis, 2020, JBI, https://synthesismanual.jbi.global.

[17] Munn Z. , Moola S. , Lisy K. , Riitano D. , and Tufanaru C. , Methodological Guidance for Systematic Reviews of Observational Epidemiological Studies Reporting Prevalence and Cumulative Incidence Data, International Journal of Evidence-Based Healthcare. (2015) 13, no. 3, 147–153, 10.1097/XEB.0000000000000054. 26317388

[18] Munn Z. , Barker T. H. , Moola S. et al., Methodological Quality of Case Series Studies: An Introduction to the JBI Critical Appraisal Tool, JBI Evidence Synthesis. (2020) 18, no. 10, 2127–2133, 10.11124/JBISRIR-D-19-00099. 33038125

[19] Changoh C. M. , Tatah L. , Aroke D. , Nsagha D. , and Choukem S. P. , Noncommunicable Diseases Behavioural Risk Factors Among Secondary School Adolescents in Urban Cameroon, BMC Public Health. (2024) 24, no. 1, 10.1186/s12889-024-17753-1. PMC10840178 38317170

[20] Bilog N. C. , Ndongo J. M. , Bika Lele E. C. et al., Prevalence of Metabolic Syndrome and Components in Rural, Semi-Urban and Urban Areas in the Littoral Region in Cameroon: Impact of Physical Activity, Journal of Health, Population and Nutrition. (2023) 42, no. 1, 10.1186/s41043-023-00415-0. PMC10496244 37697395

[21] Kamdem F. , Bika Léle E. C. , Hamadou B. et al., Prevalence and Risk Factors of Pre‐Hypertension and High Blood Pressure Among Adolescents in Cameroonian Schools, Journal of Clinical Hypertension. (2023) 25, no. 9, 845–852, 10.1111/jch.14711. 37561361 PMC10497023

[22] Mouna J. K. , Assomo Ndemba P. B. , Dalle E. et al., The Relationship Between the Physical Fitness and Academic Performance of Students in Douala, Cameroon: A Cross-Sectional Study, International Journal of School Health. (2020) 7, no. 4, 10.30476/intjsh.2020.87196.1094.

[23] Djeukeu A. W. , Kana-Sop M. M. , Gouado I. et al., Feeding Practices and Nutritional Parameters of Children Aged 6-14 Years From Cameroon, Global Journal of Medical Research. (2013) 13, no. 2.

[24] Boukeng L. B. K. , Minkandi C. A. , and Dapi L. N. , Oral Pathology and Overweight Among Pupils in Government Primary Schools in Cameroon: A Cross-Sectional Study, BMC Oral Health. (2023) 23, no. 1, 10.1186/s12903-023-02941-z. PMC10176739 37173666

[25] Yamgai F. P. , Pouokam G. B. , Saha F. B. U. et al., Combined Education Course on Nutrition, Hand-Washing and Dental Care in Primary Schools in Yaoundé, Cameroon, JoGHR. (2022) 6, 10.29392/001c.33812.

[26] Chelo D. , Mah E. M. , Chiabi E. N. et al., Prevalence and Factors Associated With Hypertension in Primary School Children, in the Centre Region of Cameroon, Translational Pediatrics. (2019) 8, no. 5, 391–397, 10.21037/tp.2019.03.02. 31993352 PMC6970110

[27] Simomia M. L. B. and Nguendo Y. H. B. , Overweight and Obesity Among School-Aged Adolescents in Yaoundé, International Journal of Public Health Research. (2019) 9, no. 1, 1059–1072.

[28] Pial A. C. , Mboupaing M. , Majesté P. , and Ndengue G. , Etat Des Lieux Du Dispositif D’alimentation Dans Quelques Écoles Primaires De La Ville De Yaoundé (Cameroun), ESJ. (2017) 13, no. 18, 10.19044/esj..v13n18p314.

[29] Said-Mohamed R. , Bernard J. Y. , Ndzana A. C. , and Pasquet P. , Is Overweight in Stunted Preschool Children in Cameroon Related to Reductions in Fat Oxidation, Resting Energy Expenditure and Physical Activity?, PLoS One. (2012) 7, no. 6, 10.1371/journal.pone.0039007. PMC3372472 22701741

[30] Dapi L. N. , Socioeconomic and Sex Differences in Adolescents’ Dietary Intake, Anthropometry and Physical Activity in Cameroon, Africa, 2010, Umeå University, Faculty of Medicine.

[31] Dapi L. N. , Janlert U. , Nouedoui C. , Stenlund H. , and Håglin L. , Socioeconomic and Gender Differences in Adolescents’ Nutritional Status in Urban Cameroon, Africa, Nutrition Research. (2009) 29, no. 5, 313–319, 10.1016/j.nutres.2009.05.002. 19555812

[32] Said-Mohamed R. , Allirot X. , Sobgui M. , and Pasquet P. , Determinants of Overweight Associated With Stunting in Preschool Children of Yaoundé, Cameroon, Annals of Human Biology. (2009) 36, no. 2, 146–161, 10.1080/03014460802660526. 19191081

[33] Dapi L. N. , Omoloko C. , Janlert U. , Dahlgren L. , and Håglin L. , ‘I Eat to Be Happy, to Be Strong, and to Live.’ Perceptions of Rural and Urban Adolescents in Cameroon, Africa, Journal of Nutrition Education and Behavior. (2007) 39, no. 6, 320–326, 10.1016/j.jneb.2007.03.001. 17996627

[34] Dapi L. N. , Nouedoui C. , Janlert U. , and Haglin L. , Adolescents’ Food Habits and Nutritional Status in Urban and Rural Areas in Cameroon, Africa, Scandinavian Journal of Nutrition. (2005) 49, 151–158, 10.1080/11026480500437554.

[35] Mirabelle Boh N. , Aba E. R. , and Burnice Lemfor C. , Dietary Practices and Nutrient Intake of Internally Displaced School Children in the West Region of Cameroon, International Journal of Food Science. (2023) 2023, 12, 9954118, 10.1155/2023/9954118. 36852392 PMC9966561

[36] Niba L. L. , Dzekem A. Y. , Navti L. K. , and Moses S. , Predictors of Herbal Medicine Use Amongst Adults With Type 2 Diabetes in an Urban Setting in Cameroon, Journal of Biosciences and Medicines. (2023) 11, no. 4, 182–198, 10.4236/jbm.114013.

[37] Navti L. K. and Foudjo B. U. S. , 10-Year Changes in Adiposity in Cameroon School-Age Children: Evidence for Increasing Central Adiposity and Higher Adiposity Levels in Tallest-for-Age Children, Journal of Obesity. (2021) 2021, 8, 6866911, 10.1155/2021/6866911. 34691777 PMC8536440

[38] Navti L. K. , Atanga M. B. , and Niba L. L. , Associations of out of School Physical Activity, Sedentary Lifestyle and Socioeconomic Status With Weight Status and Adiposity of Cameroon Children, BMC Obes. (2017) 4, no. 1, 10.1186/s40608-017-0171-3. PMC5678598 29152311

[39] Navti L. K. , Ferrari U. , Tange E. , Parhofer K. G. , and Bechtold-Dalla P. S. , Height-Obesity Relationship in School Children in Sub-Saharan Africa: Results of a Cross-Sectional Study in Cameroon, BMC Research Notes. (2015) 8, no. 1, 10.1186/s13104-015-1073-4. PMC4377213 25889151

[40] Navti L. K. , Ferrari U. , Tange E. , Bechtold-Dalla Pozza S. , and Parhofer K. G. , Contribution of Socioeconomic Status, Stature and Birth Weight to Obesity in Sub-Saharan Africa: Cross-Sectional Data from Primary School-Age Children in Cameroon, BMC Public Health. (2014) 14, no. 1, 10.1186/1471-2458-14-320. PMC3983851 24708806

[41] Sumbele I. U. N. , Teh R. N. , Nkeudem G. A. et al., Asymptomatic and Sub-Microscopic Plasmodium falciparum Infection in Children in the Mount Cameroon Area: A Cross-Sectional Study on Altitudinal Influence, Haematological Parameters and Risk Factors, Malaria Journal. (2021) 20, no. 1, 10.1186/s12936-021-03916-7. PMC8474836 34565353

[42] Sumbele I. U. N. , Nkain A. J. , Ning T. R. , Anchang-Kimbi J. K. , and Kimbi H. K. , Influence of Malaria, Soil-Transmitted Helminths and Malnutrition on Haemoglobin Level Among School-Aged Children in Muyuka, Southwest Cameroon: A Cross-Sectional Study on Outcomes, PLoS One. (2020) 15, no. 3, 10.1371/journal.pone.0230882. PMC7105131 32226023

[43] Tabi E. S. B. , Cumber S. N. , Juma K. O. , Ngoh E. A. , Achidi E. A. , and Eyong E. M. , A Cross-Sectional Survey on the Prevalence of Anemia and Malnutrition in Primary School Children in the Tiko Health District, Cameroon, Pan African Medical Journal. (2019) 32, no. 111, 5–7, 10.11604/pamj.2019.32.111.15728. PMC6560948 31223401

[44] Mbuh J. V. and Nembu N. E. , Malnutrition and Intestinal Helminth Infections in Schoolchildren From Dibanda, Cameroon, Journal of Helminthology. (2013) 87, no. 1, 46–51, 10.1017/S0022149X12000016. 22273401

[45] Marat I. M. , Loubou M. L. , Luc-Aime K. S. , Aminou M. , and Marcellin N. G. , Influence of Socioeconomic Factors on the Nutritional Status of Pupils of 5 to 11 in Rural Areas in Cameroon: Case of the Nyambaka Municipality in the Adamawa Region, Indian Journal of Public Health. (2022) 66, no. 2, 193–195, 10.4103/ijph.ijph_1516_21. 35859505

[46] Garba C. M. G. and Mbofung C. M. F. , Relationship Between Malnutrition and Parasitic Infection Amongschool Children in the Adamawa Region of Cameroon, Pakistan Journal of Nutrition. (2010) 9, no. 11, 1094–1099, 10.3923/pjn.2010.1094.1099.

[47] Collishaw A. , Snider A. , McNamara P. , Nuvaga S. , and Bilame A. F. , Impact of a Video-Based Nutrition Education Program on the Nutrition Knowledge of Students and Parents: Evidence From the North Region of Cameroon, Global Health Promotion. (2024) 31, no. 3, 60–69, 10.1177/17579759231206795. 37990146

[48] Chiabi A. , Nem D. , Kobela M. , Mbuagbaw L. , Obama M. T. , and Ekoe T. , Anthropometric Measurements of Preschool Children in North Cameroon, Eastern Journal of Medicine. (2011) 16, 240–247.

[49] OCHA (United Nations Office for the Coordination of Humanitarian Affairs) , Cameroun Rapport de Situation, 2023, https://reports.unocha.org/fr/country/cameroon/.

[50] Dama U. , Tchoffo D. , Akoa F. A. O. et al., Prévalence de la Malnutrition Chez Les Enfants De Moins De 5 Ans Dans Les Départements Du Mayo-Tsanaga Et Du Logone Et Chari, Extrême-Nord, Cameroun, PAMJ Clinical Medicine. (2024) 14, no. 3, 10.11604/pamj-cm.2024.14.3.41534.

[51] Manjong F. T. , Verla V. S. , Egbe T. O. , and Nsagha D. S. , Undernutrition Among Under-Five Indigenous Mbororo Children in the Foumban and Galim Health Districts of Cameroon: A Cross-Sectional Study, Pan African Medical Journal. (2021) 38, 10.11604/pamj.2021.38.352.25030. PMC8308853 34367431

[52] Njoya Hamza M. , Dorothy Engwali F. , and Soh W. B. D. , Assessing the Food Security of Vulnerable Agricultural Households to Climate Change in the Council of Tokombéré, far North Région of Cameroon: An Analysis Focused on the FCS, HDDS and CSI, International Journal of Agricultural Economics. (2019) 4, no. 1, 19–25, 10.11648/j.ijae.20190401.13.

[53] Khamis A. G. , Mwanri A. W. , Ntwenya J. E. , and Kreppel K. , The Influence of Dietary Diversity on the Nutritional Status of Children Between 6 and 23 Months of Age in Tanzania, BMC Pediatrics. (December 2019) 19, no. 1, 10.1186/s12887-019-1897-5. PMC6935228 31881999

[54] Dearden K. A. , Schott W. , Crookston B. T. , Humphries D. L. , Penny E. , and Behrman J. R. , Children with Access to Improved Sanitation but Not Improved Water are at Lower Risk of Stunting Compared to Children Without Access: A Cohort Study in Ethiopia, India, Peru, and Vietnam, BMC Public Health. (2017) 17, no. 1, 10.1186/s12889-017-4033-1. PMC5259877 28114914

[55] Khalid F. A. , Eldirdery M. M. , ElGasim M. E. , Elhaj M. A. , Desogi M. A. , and Mukhtar M. M. , Prevalence of Malnutrition in School Aged Children, Kassala State, Sudan, Journal of Nutrition & Food Sciences. (2021) 11, 10.21203/rs.3.rs-939475/v1.

[56] Ganle J. K. , Boakye P. P. , and Baatiema L. , Childhood Obesity in Urban Ghana: Evidence From a Cross-Sectional Survey of In-School Children Aged 5-16 Years, BMC Public Health. (2019) 19, no. 1, 10.1186/s12889-019-7898-3. PMC6880588 31771549

[57] Aboagye R. G. , Kugbey N. , Ahinkorah B. O. et al., Nutritional Status of School Children in the South Tongu District, Ghana, PLoS One. (2022) 17, no. 8, 10.1371/journal.pone.0269718. PMC9401153 36001627

[58] Aryeetey R. , Lartey A. , Marquis G. S. , Nti H. , Colecraft E. , and Brown P. , Prevalence and Predictors of Overweight and Obesity Among School-Aged Children in Urban Ghana, BMC Obesity. (2017) 4, no. 1, 1–8, 10.1186/s40608-017-0174-0. 29214030 PMC5715494

[59] Ofori-Asenso R. , Agyeman A. A. , Laar A. , and Boateng D. , Overweight and Obesity Epidemic in Ghana-A Systematic Review and Meta-Analysis, BMC Public Health. (2016) 16, no. 1, 10.1186/s12889-016-3901-4. PMC5148846 27938360

[60] Mekonnen T. , Tariku A. , and Abebe S. M. , Overweight/Obesity Among School Aged Children in Bahir Dar City: Cross Sectional Study, Italian Journal of Pediatrics. (2018) 44, no. 1, 10.1186/s13052-018-0452-6. PMC5781282 29361952

[61] Erismann S. , Knoblauch A. M. , Diagbouga S. et al., Prevalence and Risk Factors of Undernutrition Among Schoolchildren in the Plateau Central and Centre-Ouest Regions of Burkina Faso, Infectious Diseases of Poverty. (2017) 6, no. 1, 10.1186/s40249-016-0230-x. PMC5244543 28100278

[62] Adetunji A. E. , Adeniran K. A. , Olomu S. C. et al., Socio-Demographic Factors Associated With Overweight and Obesity Among Primary School Children in Semi-Urban Areas of Mid-Western Nigeria, PLoS One. (2019) 14, no. 4, 10.1371/journal.pone.0214570. PMC6447222 30943233

[63] Caulfield L. E. , Richard S. , and Black E. , Undernutrition as an Underlying Cause of Malaria Morbidity and Mortality in Children Less Than Five Years Old, American Journal of Tropical Medicine and Hygiene. (2004) 71, no. 2 Suppl, 55–63, 10.4269/ajtmh.2004.71.55. 15331819

[64] Cegielski J. P. and McMurray D. N. , The Relationship Between Malnutrition and Tuberculosis: Evidence From Studies in Humans and Experimental Animals, International Journal of Tuberculosis & Lung Disease. (2004) 8, no. 3, 286–298. 15139466

[65] World Health Organization , Improving Nutrition Outcomes With Better Water, Sanitation and Hygiene: Practical Solutions for Policies and Programmes, 2015.

[66] Strunz E. C. , Addiss D. G. , Stocks M. E. , Ogden S. , Utzinger J. , and Freeman M. C. , Water, Sanitation, Hygiene, and Soil-Transmitted Helminth Infection: A Systematic Review and Meta-Analysis, PLoS Medicine. (2014) 11, no. 3, 10.1371/journal.pmed.1001620. PMC3965411 24667810

[67] Krawinkel M. B. , Interaction of Nutrition and Infections Globally: An Overview, Annals of Nutrition & Metabolism. (2012) 61, no. 1, 39–45, 10.1159/000345162. 23343946

[68] Kimassoum D. , Alexis H. , Abdelsalam T. , Zoumaye K. , and Frazzoli C. , An Exploratory Investigation of Individual and School-Level Determinants for Effective Nutrition Education in Primary School in N’Djamena, Chad, African Journal of Food Science Research. (2022) 10, no. 3.

[69] Bain L. E. , Awah P. K. , Geraldine N. et al., Malnutrition in Sub-Saharan Africa: Burden, Causes and Prospects, PAMJ. (2013) 15, no. 1, 1–3, 10.11604/pamj.2013.15.120.2535. 24255726 PMC3830470

[70] Wardle J. , Parmenter K. , and Waller J. , Nutrition Knowledge and Food Intake, Appetite. (2000) 34, no. 3, 269–275, 10.1006/appe.1999.0311. 10888290

[71] Gewa C. A. , Childhood Overweight and Obesity Among Kenyan Pre-school Children: Association With Maternal and Early Child Nutritional Factors, Public Health Nutrition. (2010) 13, no. 4, 496–503, 10.1017/S136898000999187X. 19889248

